# Updated Genome Assembly and Annotation for *Metrosideros polymorpha*, an Emerging Model Tree Species of Ecological Divergence

**DOI:** 10.1534/g3.119.400643

**Published:** 2019-09-20

**Authors:** Ayako Izuno, Thomas Wicker, Masaomi Hatakeyama, Dario Copetti, Kentaro K. Shimizu

**Affiliations:** * Department of Forest Molecular Genetics and Biotechnology, Forestry and Forest Products Research Institute, Tsukuba, Ibaraki 305-8687, Japan,; † Department of Evolutionary Biology and Environmental Studies, University of Zurich, CH-8057 Zurich, Switzerland,; ‡ Department of Plant and Microbial Biology, University of Zurich, CH-8008 Zurich, Switzerland,; § Functional Genomics Center Zurich, CH-8057 Zurich, Switzerland,; ** Swiss Institute of Bioinformatics, CH-1015 Lausanne, Switzerland,; †† Institute of Agricultural Sciences, ETH Zurich, CH-8092 Zurich, Switzerland, and; ‡‡ Kihara Institute for Biological Research (KIBR), Yokohama City University, 641-12 Maioka, Totsuka-ward, Yokohama 244-0813, Japan

**Keywords:** Hawaii, MAKER, *Metrosideros polymorpha*, reannotation, transposable element

## Abstract

Accurate feature annotation as well as assembly contiguity are important requisites of a modern genome assembly. They allow large-scale comparison of genomes across and within species and identification of polymorphisms, leading evolutionary and functional studies. We report an updated genome resource for *Metrosideros polymorpha*, the most dominant tree species in the Hawaiian native forests and a unique example of rapid and remarkable ecological diversification of woody species. Ninety-one percent of the bases in the sequence assembly (304 Mb) were organized into 11 pseudo-molecules, which would represent the chromosome structure of the species assuming the synteny to a close relative *Eucalyptus*. Our complementary approach using manual annotation and automated pipelines identified 11.30% of the assembly to be transposable elements, in contrast to 4.1% in previous automated annotation. By increasing transcript and protein sequence data, we predicted 27,620 gene models with high concordance from the supplied evidence. We believe that this assembly, improved for contiguity, and annotation will be valuable for future evolutionary studies of *M. polymorpha* and closely related species, facilitating the isolation of specific genes and the investigation of genome-wide polymorphisms associated with ecological divergence.

*Metrosideros polymorpha* Gaud. (Myrtaceae) represents a unique example of rapid and remarkable ecological diversification of woody species. This species has dominated the diverse habitats ranging over 2,000 m in altitude since the colonization on the Hawaiian Islands in 1–3.9 million years ago (Wright *et al.* 2000; Percy *et al.* 2008). As eight varieties are recognized (Dawson and Stemmermann 1990), plants from different environment or successional stages show distinct morphological (*e.g.*, Stemmermann 1983; Joel *et al.* 1994; Tsujii *et al.* 2016), chemical (*e.g.*, Vitousek *et al.* 1988, 1992), and physiological (*e.g.*, Cordell *et al.* 1998; Cornwell *et al.* 2007) characteristics. Despite of the ecological differences, the reproductive isolation is incomplete (Stacy *et al.* 2016, 2017) and gene flow occurs between populations in different environments (DeBoer and Stacy 2013; Stacy *et al.* 2014; Izuno *et al.* 2017). Therefore, *M. polymorpha* can serve as a model to study the early stages of speciation of tree species.

The first genome assembly of *M. polymorpha* (version 1.0 hereon) was reported as 304 Mb sequences, half of which were composed of 19 scaffolds with >5 Mbp (Izuno *et al.* 2016). On the assembly, RepeatMasker (Smit *et al.* 2013) identified 4.1% of the bases to be associated with repeated sequences and transposable elements (TEs), based on the homology with *Arabidopsis thaliana* repeat sequences. AUGUSTUS (Stanke *et al.* 2004) predicted 39,305 gene models based on a Hidden Markov model trained with RNA-seq data from one *M. polymorpha* plant. Although this genome resource was useful to obtain genomic insights of the ecological diversification in the species (Izuno *et al.* 2016, 2017), there was still room for improvement in the genome resource. Slightly more number of gene models (39,305) were identified compared to other tree genomes (approximately 33,000 genes on average; Neale *et al.* 2017), indicating that some gene models could be fragmented. The annotated portion of transposable elements was relatively low (4.1%), considering other tree genomes with similar genome size (300–350 Mb) contain transposable elements in 20–30% of the whole genomes (He *et al.* 2013; Verde *et al.* 2013; Wu *et al.* 2014). This suggests that *M. polymorpha* probably contained additional TE/repeat regions that escaped detection.

Here, we report the updated version of the *M. polymorpha* genome sequences (version 2.0 hereon). It was obtained by assigning the ver. 1.0 assembly (36,375 scaffolds) to 11 pseudo-chromosomes by means of the presumed collinearity with the closely related *Eucalyptus grandis*. To improve the quality of gene prediction, we used an increased number of evidence data, such as RNA-Seq and protein sequences data, to train gene prediction programs. We manually annotated repeat regions in addition to using automatic pipelines to gather repeat sequences that were not identified in the ver. 1.0 annotation. The resulting ver. 2.0 annotation contained more complete gene models that were well supported by supplied evidence data and showed that an increased, but still small, fraction of the genomes is composed by TEs and repeated sequences.

## Materials and Methods

### Pseudo-molecule assembly

*Metrosideros polymorpha* pseudo-molecules were constructed based on the *Eucalyptus grandi*s genome (ver. 2.0; Bartholomé *et al.* 2014), which is composed of 11 pseudo-chromosomes and 4,932 scaffolds. We assumed chromosome structure be mostly conserved between the two species because the basic chromosome number for Myrtaceae is *x* = 11 (Atchison 1947) and *M. polymorpha* also has 11 chromosomes (2*n* = 22; Carr 1978). We identified the positions of 32,152 *E. grandis* coding DNA sequences (CDSs) on the 36,376 *M. polymorpha* scaffolds (ver. 1.0) by NCBI BLASTN (ver. 2.2.31+) with the threshold cutoff of 1.0E-3. *Metrosideros polymorpha* scaffolds with more than 10 CDSs were used for anchoring to *E. grandis* pseudo-chromosomes. For all CDSs on these scaffolds, it was first determined to which *E. grandis* chromosome they map. Then, we removed CDSs that map to different *E. grandis* chromosomes than the majority of CDS on the scaffold or to very distant regions of the same *E. grandis* chromosome. This step removed approximately 12% of the CDS (a low value due to good collinearity between *E. grandis* and *M. polymorpha*). To determine a single anchor point for each scaffold, the average position of the central five CDSs on the corresponding *E. grandis* chromosome was used. These anchor points were then used to determine the linear order of *M. polymorpha* scaffolds in the pseudo-chromosomes. Finally, *M. polymorpha* scaffolds were oriented based on the gene order in *E. grandis* pseudo-chromosomes.

### Manual annotation for transposable elements (TEs)

We produced a hand-curated reference TE library for *M. polymorpha*. For *Gypsy*, *Copia*, *CACTA*, *hAT* and *Helitron* superfamilies, we used predicted protein sequences from TEs previously identified in other plant species to search putative TE regions in the *M. polymorpha* pseudo-molecules using TBLASTN (ver. 2.2.31+). Predicted TE proteins were obtained from the TREP database (botinst.uzh.ch/en/research/genetics/thomasWicker/trep-db.html). Regions that showed homology to TE proteins were then extracted with 5000–9000 bp of flanking regions using an in-house script. This search produced a library of gene-bearing TEs. Subsequent BLASTN searches for sequences with DNA homology were used to identify non-autonomous deletion derivatives (*i.e.*, insertions that lack parts or all of the coding regions). Multiple sequences from individual families were used to generate a consensus sequence, which was added to the reference TE library. We further *de novo* identified DNA transposons by searching terminal inverted repeats (TIRs). Candidate TIR elements were then used in BLASTN searches against the whole genome. Those that occurred in multiple copies were considered novel DNA transposon families. For TE annotation, the identified reference TE families were mapped back to the genome using an in-house TE annotation pipeline.

### Genome annotation

**Figure 1:**
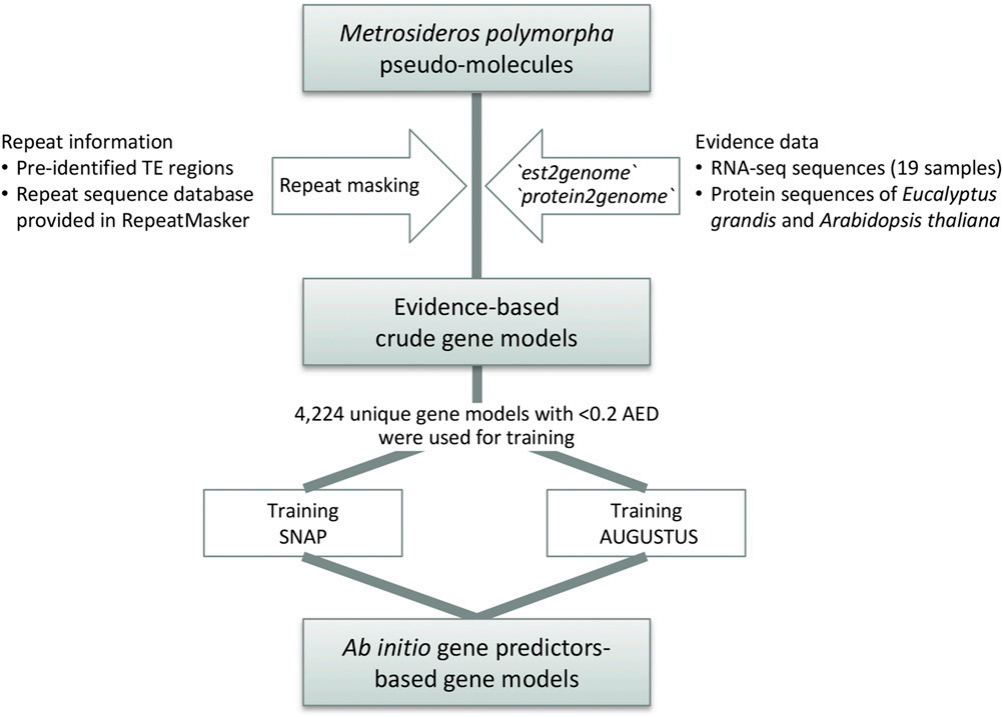
Workflow for re-annotation of the *Metrosideros polymorpha* genome using the MAKER2 pipeline.

We used the MAKER genome annotation pipeline (ver. 2.31.8; Holt and Yandell 2011) to identify gene models on the *M. polymorpha* genome ver. 2.0 (Figure 1). MAKER was initially run to make crude gene models based on transcript and protein evidence. RNA-Seq evidence, consisted of leaf bud samples from 19 different *M. polymorpha* trees at the Volcano Agriculture Station, University of Hawaii (DDBJ accession DRA00791). After trimming adapter sequences and bases with low quality using Trimmomatic (ver. 0.33; Bolger *et al.* 2014), the reads were mapped on the reference genome ver. 2.0 using STAR (ver. 2.5.1b; Dobin *et al.* 2013). The alignments were converted to transcript models into GFF format using StringTie (ver. 1.3.3; Pertea *et al.* 2015). Protein evidence was supplied from 46,280 isoform sequences of *E. grandis* (ver. 2.0; http://genome.jgi.doe.gov/pages/dynamicOrganismDownload.jsf?organism=Egrandis) and 35,386 isoform sequences of *A. thaliana* (TAIR10; http://genome.jgi.doe.gov/pages/dynamicOrganismDownload.jsf?organism=Athaliana). In the first run, EST and protein evidence were used for the gene prediction, *i.e.*, `est2genome` and `protein2genome` were set to 1 in the MAKER configuration. At the same time, we identified repeat regions using RepeatMasker (ver. 4.0.5; Smit *et al.* 2013) and RepeatRunner (Smith *et al.* 2007) with the RepBase database for ‘all species’. The TE regions manually identified (see above) were supplied as external annotations and masked during the run.

Of the 22,960 gene models generated in the initial MAKER run, 4,224 unique gene models with less than 0.2 of annotation edit distance (AED; Eilbeck *et al.* 2009) were used to train the Hidden Markov model (HMM) for SNAP (ver. 2013-02-16; Korf 2004) and AUGUSTUS (Stanke *et al.* 2004) following Campbell *et al.* (2014) and Bowman *et al.* (2017). MAKER was run again to conduct SNAP- and AUGUSTUS-based gene prediction with the trained HMMs. In the second run, we allowed predicted genes with more than 50 amino acids, and `keep_preds` and `always_complete` were set to 1.

To compare annotation quality between the current and previous genome annotations, we added MAKER’s quality-control metrics to the ver. 1.0 annotation. Using MAKER, we calculated AED scores for 41,874 mRNAs in the ver. 1.0 annotation against the evidence data used for the current annotation (obtained with the 19 RNA-seq data and protein sequences of *E. grandis* and *A. thaliana*).

For the functional annotations, the predicted protein sequences were searched among 16,712 protein domains in the Pfam (ver. 31.0; Finn *et al.* 2016) using InterProScan (ver. 5.26-65.0; Jones *et al.* 2014) as well as 44,240 protein isoform sequences for plants in the SwissProt (downloaded on January 18, 2018; The UniProt Consortium 2017) using NCBI BLASTP (ver. 2.2.31+). After removing genes with TE-related annotations, we selected gene models that were supported by transcript evidence or Pfam protein domains (*i.e.*, the ‘standard build’ in the MAKER pipeline was adopted) according to the criteria in Holt & Yandell (2011) and Campbell *et al.* (2014). The completeness of the obtained transcript isoforms among transcripts of the 1,440 single-copy orthologs universally found in plants was assessed with BUSCO (ver. 2.1; Simão *et al.* 2015) on gVolante (ver. 1.1.0; Nishimura *et al.* 2017). As a comparison, the 43,894 isoforms located in the 11 chromosomes of the *E. grandis* (ver. 2.0) were run on BUSCO as well.

### Data availability

The genome assembly (ver. 2.0) was deposited at DDBJ (BCNH02000001–BCNH02000011). The genome annotation (GFF format) is available in File S1, which is deposited at Figshare. Supplemental material available at FigShare: https://doi.org/10.6084/m9.figshare.9876644 and https://doi.org/10.6084/m9.figshare.9876659.

## Results and Discussion

### Pseudo-molecule assembly

**Figure 2:**
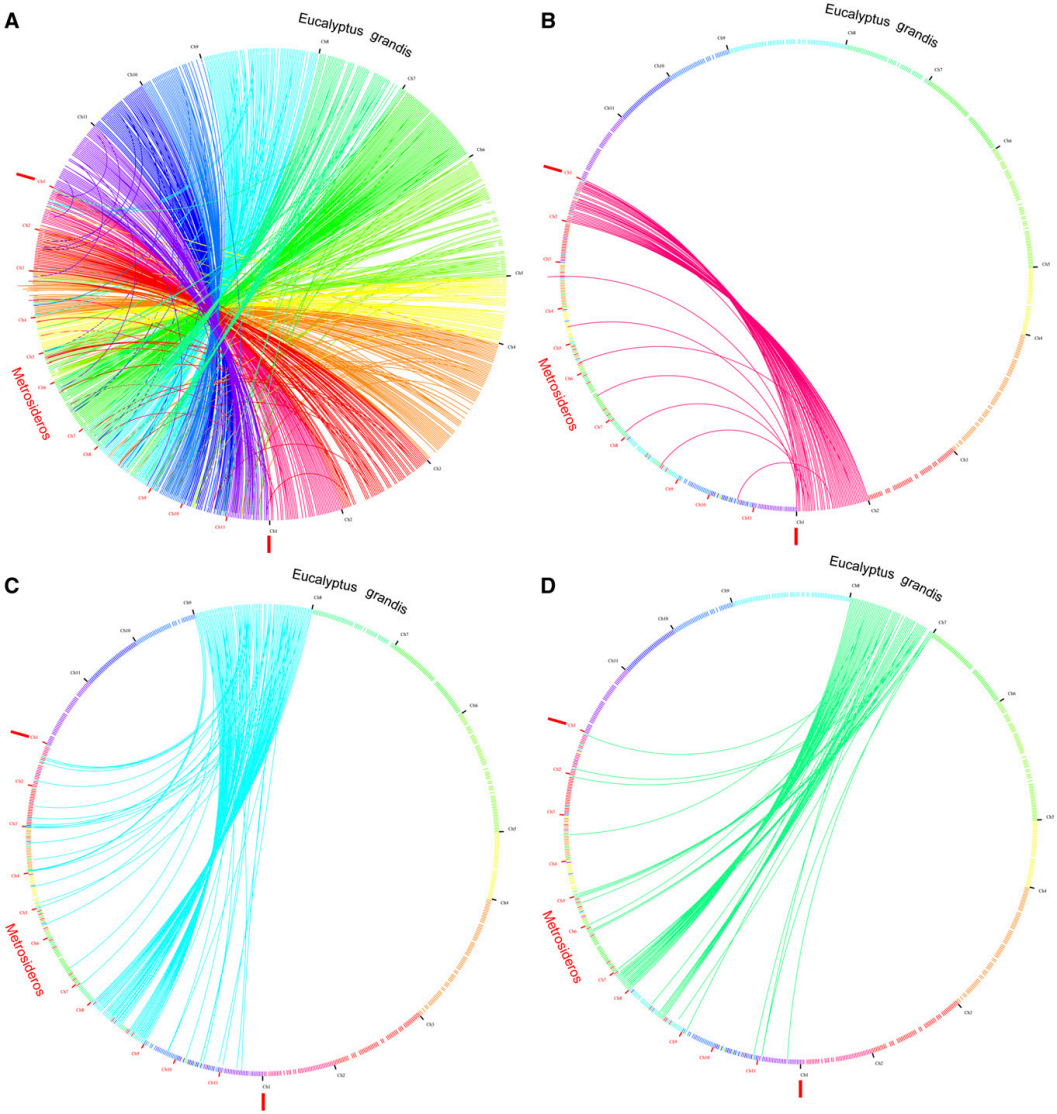
Circos plots for the comparison between *Metrosideros polymorpha* pseudo-molecules (ver. 2.0; red labels) and *Eucalyptus grandis* genome (Bartholomé *et al.* 2014; black labels). To make the plot clearer, only every 5^th^ gene is shown. (a) genome wide comparison. (b) an example of chromosome 1 which shows almost perfect conservation in gene order. (c), (d) translocations (or assembly errors) occurred between chromosomes 7 and 8.

Of the 32,152 *E. grandis* genes, 28,974 had significant homology on 424 *M. polymorpha* scaffolds. By anchoring the 102 scaffolds with more than 10 CDSs to the 11 *E. grandis* pseudo-chromosomes, we obtained 11 *M. polymorpha* pseudo-molecules ranging in size from 14.5 to 40.2 Mbp (Table 1; Figure 2; DDBJ accession BCNH02000001–BCNH02000011), which constitute 91.1% of the entire *M. polymorpha* genome assembly. This result indicated >90% of the ver. 1.0 assembly represented non-redundant regions of the genome, against our previous speculation that the relatively high heterozygosity could split the assembly. Long read sequencing technologies could align the remaining ∼10% of the scaffolds, which were short (an average of 571 bp in length) and repeat-rich (data not shown), and clarify how the complexity of genome affects *de novo* assembly.

**Table 1:** Summary of *Metrosideros polymorpha* pseudo-molecules. Sequences were assigned to 11 chromosomes based on the collinearity with *Eucalyptus* genes, assuming a complete synteny between the two species

Pseudo-molecule ID	Size, bp	Gaps, %
Mpol_Chr01	26,760,463	2.5
Mpol_Chr02	25,476,500	2.9
Mpol_Chr03	27,763,088	3.8
Mpol_Chr04	21,740,955	2.7
Mpol_Chr05	19,091,012	4.9
Mpol_Chr06	32,945,760	2.8
Mpol_Chr07	14,555,926	3.4
Mpol_Chr08	40,261,496	3.4
Mpol_Chr09	21,463,900	2.8
Mpol_Chr10	27,804,677	2.4
Mpol_Chr11	25,789,789	3.5
Total	283,653,566	3.6

The current assembly (pseudo-molecules) highlighted highly conserved gene content between the two species. After diverging from a common ancestor in the Paleocene (66–55 million years ago), *Eucalyptus* diversified in Oligocene (38–24 million years ago) on the Australia continent whereas *Metrosideros* spread throughout the Pacific islands in the Miocene (23.3–5.2 million years ago) (Thornhill *et al.* 2015; Wright *et al.* 2000). The independent evolution of the two species after split could have led to the accumulation of TEs in *Eucalyptus*, explaining the different genome size between *E. grandis* (640 Mb; Myburg *et al.* 2014) and *M. polymorpha* (∼330 Mb).

### TE annotation

**Figure 3:**
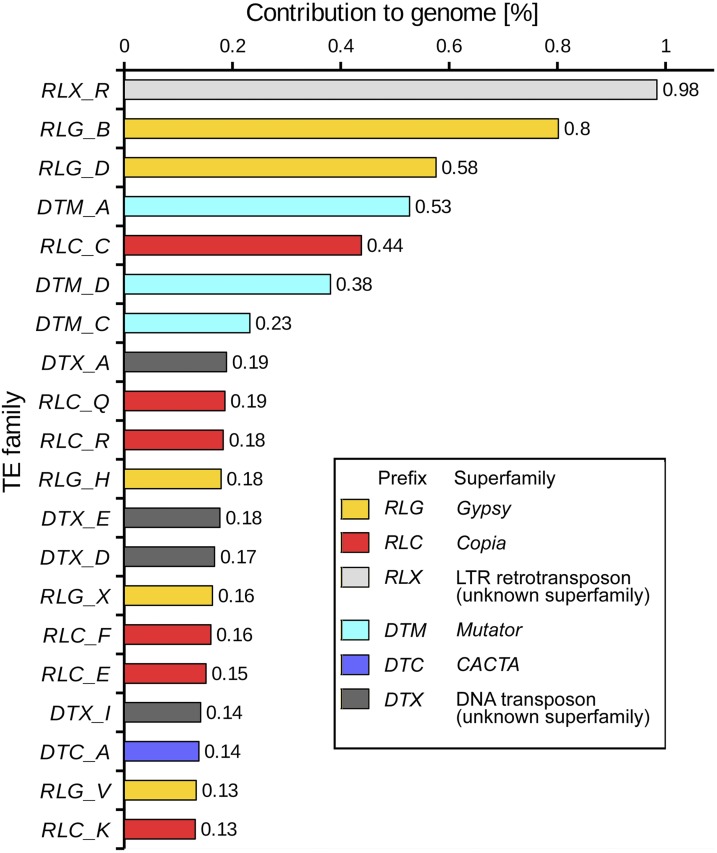
Contributions of the 20 most abundant transposable element (TE) families to the whole genome. Fifteen families could be assigned to four different superfamilies (see inset), the remaining did not contain coding sequences (*e.g.*, transposase), which would have allowed their classification into known superfamilies.

Our manual annotation identified 8.03% of the *M. polymorpha* genome (ver. 2.0) to be comprised of TEs (Table 2). Most of them were derived from *Gypsy* (2.46%) and *Copia* (2.21%) long terminal repeat retrotransposons (LTR-RTs) (Figure 3), of which 19 and 24 families were identified, respectively. DNA transposons constituted 2.43% of the genome (Table 2). We identified between one and 4 families of *Mutator*, *CACTA*, *hAT* and *Helitron* based on the homology search approach and 9 families based on the features of TIRs (Table 2). Different TE families were enriched in different regions on pseudo-molecules (Figures S1–S4), suggesting different proliferation strategies among TE families. RepeatMasker annotated 2.88% of the genome to be TEs (Table 2) and RepeatRunner identified 1,469 additional TEs spanning 0.28% of the genome. Gene annotation with MAKER further found 147 genes (spanning 0.20% of the genome) bearing TEs. With only 0.09% of the bases being identified by two or more of the annotations above, 11.30% of the assembly was characterized as TE related.

**Table 2:** Summary of transposable elements in the *Metrosideros polymorpha* pseudo-molecules identified with manual annotation and RepeatMasker

	Manual annotation				RepeatMasker		
	Nr. families	Nr. elements	TE space, bp	TE space, %	Nr. elements	TE space, bp	TE space, %
Class I (retrotransposons)							
LTR	45	30516	15896123	5.60	14095	6370191	2.25
* Gypsy*	19	15563	6978439	2.46	3769	1439715	0.51
* Copia*	24	8733	6258363	2.21	7799	4228467	1.49
* Pao*					185	21334	0.01
SINE					196	17591	0.01
LINE					2901	701030	0.25
* L1*					2020	583118	0.21
* RTE*					327	66310	0.02
* R1/LOA/Jockey*					153	19698	0.01
* L2/CR1/Rex*					175	14276	0.01
Class II (DNA transposons) Subclass 1							
TIR	16	40813	6643207	2.34	5686	820749	0.29
*Mutator*	4	22608	3564813	1.26			
*CACTA*	1	348	369394	0.13			
*hAT*	2	1396	370560	0.13	1312	199320	0.07
*PIF-Harbinger*					823	178858	0.06
*TcMar*					113	7463	<0.01
Class II (DNA transposons) Subclass 2							
*Helitron*	3	2344	238737	0.08	971	237019	0.08
Unclassified					109	10297	<0.01
Total TEs			22778067	8.03		14763005	2.88

The three annotation approaches, *i.e.*, manual annotation, RepeatMasker and RepeatRunner, complementary identified TEs in the *M. polymorpha* genome. Our manual annotation identified DNA transposons occupying 2.43% of the *M. polymorpha* genome, whereas RepeatMasker suggested much lower levels (0.37%; Table 2). While our manual annotation exhaustively identified the distributions of frequent TE superfamilies (>1% in TE space), RepeatMasker could be effective to identify rare TEs, which were not handled in our manual annotation because of the insufficient number of sequences to generate representative elements. RepeatRunner identified completely different TE features from those by RepeatMasker or our manual annotation except for one TE feature. These findings indicate that TE annotation in a novel genome, for which no useful libraries of close relatives are available, can profit from complementary approaches, such as manual identification of TE families and automated annotation using software (e.g., RepeatMasker).

In some cases, the uneven distribution of TE superfamilies along chromosomes was diagnostic to infer the location of the centromeres. In most small plant genomes, LTR-RTs are enriched in centromeric and paracentromeric regions, while non-LTR RTs and DNA transposons (more often associated with genes) are found mostly in distal chromosomal regions (Bennetzen and Wang 2014). Thus, we analyzed the abundance of DNA transposons and LTR-RTs in the *M. polymorpha* pseudomolecules. We found that LTR-RTs are indeed enriched in distinct regions (Figures S1–S4). In particular, the *Gypsy* family *RLG_V* has very narrow distributions (*i.e.*, it is found only in very narrow windows of 1–3 Mb in size while it is practically absent from all other chromosomal regions; Figures S1–S4). This distribution is reminiscent of that of *RLG_Cereba* in wheat (Wicker *et al.* 2018) and CRG retroelement in cotton (Luo *et al.* 2012), which are found almost exclusively in centromeric regions. Interestingly, some of the pseudomolecules (1, 7, 8 and 10) contain two regions that are enriched in *RLG_V* elements and other LTR-RTs (Figures S1–S4). Aware of the caveat that *M. polymorpha* pseudomolecules were built based on collinearity with *E. grandis* chromosomes, the presence of multiple regions enriched in potential centromeric LTR-RTs may highlight orientation issues or suggest that the ver. 1.0 assembly included redundant scaffolds due to heterozygosity. Alternatively, one of the two regions may be a non-functional centromere (either being recently inactivated or gaining function). Additional sequencing with long reads or DNA physical/optical mapping techniques will be necessary to build more reliable pseudomolecules.

Compared to other tree genomes sequenced (reviewed in Neale *et al.* 2017), the *M. polymorpha* genome is small and contains only a small fraction of TEs. Interestingly, other plants that are adapted to extreme conditions such as mangrove trees (Lyu *et al.* 2018), a carnivorous plant (Ibarra-Laclette *et al.* 2013) or the Antarctic midge (Kelley *et al.* 2014) were reported to have small genomes with low TE content. Thus, the small genome of *M. polymorpha* could also be the result of convergent evolution due to similar selective constraints that act on *M. polymorpha* plants when they populate their extreme and diverse environments.

### Improved gene annotation

The final MAKER run, in which SNAP and AUGUSTUS were run with custom-trained HMMs, predicted 27,620 gene models putatively coding 40,206 different transcript isoforms (Table 3; File S1). While the assembly fraction occupied by coding regions was almost the same between ver. 1.0 and ver. 2.0, the number of gene models decreased and intron sizes increased in ver. 2.0 (Table 3), suggesting fragmented gene models in ver. 1.0 were concatenated. A sharp increase in mean gene size (3.37 Kb *vs.* 4.94 Kb in ver. 1.0 and ver. 2.0, respectively) accompanied to a negligible increase in total size of the coding regions (132 and 136 Mb, respectively) validate this observation. This may be due to genes that were split in two ver. 1.0 scaffolds or to the increased supplies of evidence data.

**Table 3:** Summary of the ver. 1.0 and ver. 2.0 *Metrosideros polymorpha* genome annotations

	ver. 1.0	ver. 2.0
Nr. protein-coding genes	39305	27620
Total gene space, Mb	132.5	136.5
Gene space, %	38.2	39.3
Mean gene size, bp	3371.9	4942.7
Nr. exons per gene	5.7	6.1
Mean exon size, bp	280.2	287.5
Mean intron size, bp	420.3	683.4
Total Nr. transcript isoforms	41874	40206
Average Nr. transcript isoforms per gene	1.1	1.5
Mean coding sequence length, bp	3514.2	6860.8
Transcript isoforms with Pfam domain, %	60.8	72.0
Transcript isoforms with BLASTP hit, %	55.5	69.8
Transcript isoforms with AED < 0.5, %	66.5	88.4
Transcript isoforms with AED = 1.0, %	20.9	3.4
BUSCO complete, %	92.9	90.3
BUSCO partial, %	3.1	4.0
BUSCO missing, %	4.0	5.7

**Figure 4:**
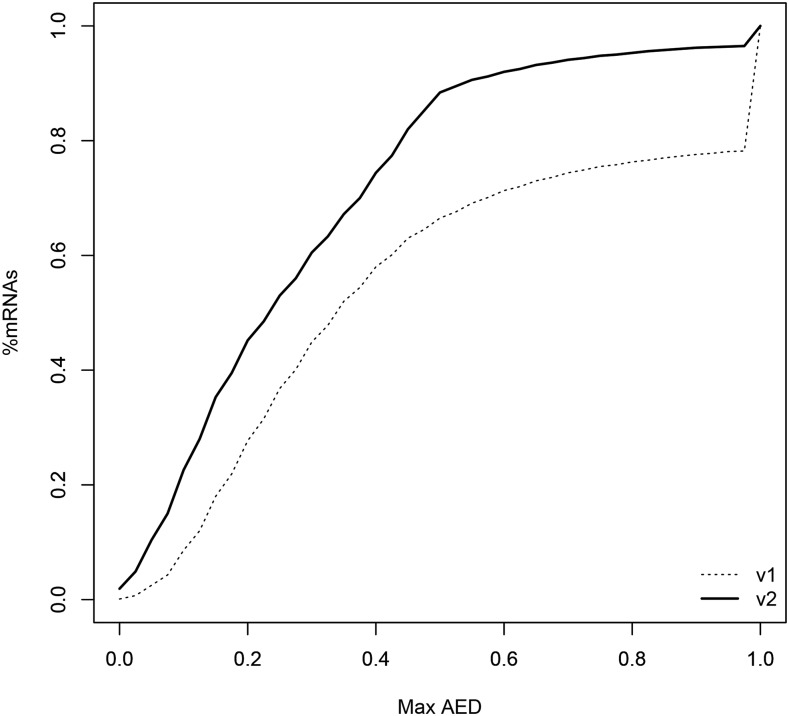
Cumulative fraction of transcript isoforms in the ver. 1.0 and ver. 2.0 *Metrosideros polymorpha* genome annotation with evidence support represented by the annotation edit distance (AED) metric. Lower AED scores indicate greater concordance with available evidence data (Eilbeck *et al.* 2009).

The concordance of the input evidence into the current gene annotation was improved: 66.5% and 88.4% transcript isoforms showed AED < 0.5 and 20.9% and 3.4% isoforms showed AED = 1 in ver. 1.0 and ver. 2.0, respectively (Figure 4). Higher proportion of isoforms in ver. 2.0 was supported by the Pfam protein domains and SwissProt proteins in comparison with ver. 1.0, possibly due to the increased length of isoforms in ver. 2.0 (Table 3). The 40,206 transcript isoforms represented ∼90% of the core transcripts from the 1,440 single-copy conserved orthologs in plants (Table 3). Although this was comparable to the completeness of the 43,894 isoforms in the 11 *E. grandis* chromosomes, which covered 92.1% of the core genes, increased evidence data from other plant tissues besides leaf buds may predict more comprehensive gene models. Overall, with increased effort on manual annotation of TEs and the increased number of evidence data, we considerably improved the quality of the *Metrosideros* gene models. This will enable more sound and complete evolutionary studies on functional genes relevant to the environmental adaptation of woody species.
